# Drivers and Socioeconomic Impacts of Tourism Participation in Protected Areas

**DOI:** 10.1371/journal.pone.0035420

**Published:** 2012-04-25

**Authors:** Wei Liu, Christine A. Vogt, Junyan Luo, Guangming He, Kenneth A. Frank, Jianguo Liu

**Affiliations:** 1 Center for Systems Integration and Sustainability, Department of Fisheries and Wildlife, Michigan State University, East Lansing, Michigan, United States of America; 2 Department of Community, Agriculture, Recreation and Resource Studies, Michigan State University, East Lansing, Michigan, United States of America; 3 Department of Counseling, Educational Psychology and Special Education, Michigan State University, East Lansing, Michigan, United States of America; Leibniz Center for Tropical Marine Ecology, Germany

## Abstract

Nature-based tourism has the potential to enhance global biodiversity conservation by providing alternative livelihood strategies for local people, which may alleviate poverty in and around protected areas. Despite the popularity of the concept of nature-based tourism as an integrated conservation and development tool, empirical research on its actual socioeconomic benefits, on the distributional pattern of these benefits, and on its direct driving factors is lacking, because relevant long-term data are rarely available. In a multi-year study in Wolong Nature Reserve, China, we followed a representative sample of 220 local households from 1999 to 2007 to investigate the diverse benefits that these households received from recent development of nature-based tourism in the area. Within eight years, the number of households directly participating in tourism activities increased from nine to sixty. In addition, about two-thirds of the other households received indirect financial benefits from tourism. We constructed an empirical household economic model to identify the factors that led to household-level participation in tourism. The results reveal the effects of local households' livelihood assets (i.e., financial, human, natural, physical, and social capitals) on the likelihood to participate directly in tourism. In general, households with greater financial (e.g., income), physical (e.g., access to key tourism sites), human (e.g., education), and social (e.g., kinship with local government officials) capitals and less natural capital (e.g., cropland) were more likely to participate in tourism activities. We found that residents in households participating in tourism tended to perceive more non-financial benefits in addition to more negative environmental impacts of tourism compared with households not participating in tourism. These findings suggest that socioeconomic impact analysis and change monitoring should be included in nature-based tourism management systems for long-term sustainability of protected areas.

## Introduction

Establishing protected areas is among the major strategies for stemming the rapid loss of global biodiversity. Over the last half century the total coverage of protected areas worldwide has increased by ten-fold whereas the trend in global biodiversity loss continues [1], [2]. While protected areas will continue to play an important role in conservation [3], the classic “fine and fence” method of management, which regards local people as a direct threat to biodiversity, has gradually given way to new approaches, such as the integrated conservation and development projects (ICDP) and payment for ecosystem service (PES) programs [4], [5], [6], [7]. These new approaches recognize the trade-offs and linkages between human livelihood and biodiversity conservation. They focus on involving local communities in conservation, and use market tools to add economic value to biodiversity [4], [5], [8], [9]. These approaches suggest that providing alternative sources of income to local communities through new livelihood opportunities or direct payments will help alleviate poverty and improve environmental awareness and conservation attitudes, which may eventually change the unsustainable resource extraction behaviors of local people and reduce human pressure on natural systems [10], [11], [12].

Although the new approaches have gradually become mainstream concepts in conservation programs, there is still a lack of convincing empirical evidence that they are effective in achieving desired and balanced social, economic, and ecological goals [4], [13]. For instance, many ICDPs fall short in creating enough incentives to discourage human activities that threaten biodiversity. In cases where desired economic benefits were indeed generated, they were often enjoyed by a few local elites or siphoned to outside investors, whereas the poorest members of the community remain marginalized [9], [14], [15]. These issues are at least partly due to over-simplified assumptions about targeted local communities in the management approach. Although there is always heterogeneity and complexity in communities, ICDPs often conceptualize them as small, homogenous, and static. Moreover, these communities are characterized as being unable or ill-equipped to succeed when new economic opportunities are offered [4]. To transform or evolve an entire targeted population, it is important to understand first how social and economic differentiation within a community, such as variation in the quantity and structure of livelihood assets owned by different households, affects community members' capacity to participate in an ICDP [16], [17].

Livelihood assets refer to the capital endowments owned by a household and include financial, human, natural, physical, and social capital [18], [19]. Financial capital refers to savings, credit and income; human capital refers to the education, skills, knowledge, and the ability household members to work; natural capital refers to natural resources owned by a household such as land, forests and fisheries; physical capital refers to a household's access to basic infrastructure, such as roads and schools, and tools and equipment; and social capital refers to the social resources of the household, such as membership in organizations and “connections” to others in power.

Different assets are required to achieve different livelihood goals. Households with more assets tend to be more versatile in choosing livelihood strategies [18]. Diversifying livelihood and income sources has been common for rural households across the developing world [20], [21]. Livelihood diversification by households is conceptualized as a process whereby labor supply and capital investment are distributed among farm, off-farm and non-farm activities within a local or regional economy. Households aim to maximize earnings subject to constraints imposed by limited capital resources in a trade-off with the desire to minimize risk [22]. We hypothesize that if an ICDP offers desired income-generating opportunities to a community, the choice by a household to participate in the project is largely affected by the family's livelihood assets.

Nature-based tourism is an important ecosystem service and one key activity in which rural households in developing countries can engage with and which has been used pervasively in ICDPs [11], [23], [24]. Tourism is arguably the world's largest industry, and nature-based tourism (also often called ecotourism, although this term actually refers to a subset of nature-based tourism activities [25]) is the fastest growing segment of the tourism industry [26]. Nature-based tourism has great potential to improve biodiversity conservation and reduce poverty [24], [27], [28] compared to other economic development options in and around protected areas for the following reasons. First, tourism is a labor-intensive industry and has the potential to create more jobs per unit of investment than most other industries. In addition, tourism can be a useful source of employment for traditionally marginalized group, including women and ethnic minorities. Second, tourism is widely perceived to be “clean”, “non-consumptive”, and inexpensive to develop because of its use of existing natural, cultural, and historical resources. Third, tourism can attract outside investments in the development of the infrastructure, including roads and public services in the destination area, which can serve the needs of both local people and tourists. Fourth, nature-based tourism draws on local knowledge, a form of human capital possessed by local households. When developing tourism activities, interactions between service providers (locals) and receivers (tourists) take place and leave important social impacts and potential benefits. Finally, specifically in developing countries, nature-based tourism should generate jobs and income opportunities for local communities, as well as help finance conservation, through government and non-government programs and the tourists themselves [29], [30].

Despite all the promises, in practice nature-based tourism sometimes results in significant negative environmental and socioeconomic impacts. The lack of local community involvement was found to be one of the top reasons behind such failures [31]. The long-term sustainability of nature-based tourism in and near protected areas is strongly dependent on its ability to improve the livelihood of local communities and to enhance local residents' attitudes and behaviors toward conservation. From a development perspective, tourism is successful only if the majority of the local community is involved and if it receives benefits equitably. From a conservation perspective, tourism is successful if the poor are preferentially targeted with jobs and poverty is reduced [27], [32], [33], [34]. However, opportunities for local participation in tourism are not always equally accessible to all community members [23], [27], [35], [36]. The skill sets demanded by tourism jobs are typically not possessed by rural residents [35]. There are other barriers, such as the distance of residence to key tourism sites, hygiene, lack of social status and family connections, and lack of start-up capital, that prevent local residents from working in and owning businesses in the tourism industry [36]. As a result, the benefits of tourism development often accrue to a few local elites and rarely reach the poor [23], [27], [36].

To better understand the role that tourism plays in biodiversity conservation, systematic research with empirical data and quantitative analysis on the various direct and indirect financial and non-financial benefits and impacts that tourism brings to local communities in and near protected areas is needed [23], [37]. Ideally, such studies should follow communities during tourism development to collect baseline and subsequent monitoring data, so that longitudinal comparisons can be made. The tourism area life cycle theory, one of the best known theories on the evolution of tourism destinations [16], [17], offers a relevant framework in terms of identifying development milestones for monitoring changes resulting from tourism development. According to tourism area life cycle theory, development of a tourism destination follows a succession of phases from exploration and involvement stages to development and maturity stages. A tourism destination in the exploration stage is characterized by a small number of tourists, an irregular pattern of visitations, and a lack of specific tourism facilities. As visitation increases and follows some regularity, the local community starts to develop specific tourism facilities and the destination enters the involvement stage. In the development stage tourist volume continues to increase and the destination becomes fully developed. In this stage local control of tourism development may start to weaken rapidly and new facilities provided by outside organizations looking for high-volume businesses gradually dominate the market. When the increasing rate of visitation starts to decline and tourist volume reaches a peak, the maturity stage is reached. In other words, tourism stops growing. Following the maturity stage is either a decline stage, in which tourist volume goes down, or a rejuvenation stage, in which new attractions are developed and visitation goes up again [16], [17]. Despite the popularity and the amount of funding invested in nature-based tourism development and conservation, nature-based tourism in protected areas of developing countries has rarely been studied under a tourism area life cycle framework [38] and the socioeconomic impacts of nature-based tourism through multiple stages have not been analyzed.

The aim of the study was to examine the nature, extent, and drivers of local households' participation in, and benefiting from, nature-based tourism in a biosphere reserve during a period of fast economic transition from agriculture and natural resources extraction to tourism. Applying the tourism area life theory, we identified various types of direct and indirect financial and non-financial benefits from tourism development over multiple life stages and modeled the determinants of household-level tourism participation. This study expands the understanding of the diverse socioeconomic impacts of tourism in protected areas.

The specific objectives of the study were to (1) enrich the conservation literature with longitudinal analysis of residents' participating and benefiting from tourism in protected areas, (2) demonstrate that livelihood assets can be a valid predictor of households' likelihood of tourism participation, (3) illustrate the relationship between tourism participation and local residents' environmental awareness and conservation attitudes, and (4) provide protected area managers with useful policy options that may encourage and facilitate more tourism participation at local levels to assist rural residents and to enhance biodiversity conservation. From these objectives, three research questions emerged:

1. What are the various ways that local residents participate in and benefit from tourism?
2. How do the quantity and structure of livelihood assets owned by local households affect their likelihood to participate in tourism?
3. Do people in tourism-participating households have particular perceptions of the socioeconomic and environmental impacts of tourism development?

## Methods

### Ethics statement

Permission from the Wolong Administration Bureau was sought and obtained before the individual subjects were contacted. Because many adult subjects were not literate, a verbal consent process was used. A verbal consent script was read to the subjects. Interviews proceeded only after the subjects gave their verbal consent. In case of non-consent, no further information was recorded. Because signed consent forms constitute a possible source of concern for the protection of respondents' confidentiality, signatures were collected during the verbal consent process. The study, including the verbal consent process and script, was reviewed and approved by the Institutional Review Board (IRB) of the Michigan State University (http://www.humanresearch.msu.edu/).

### Study area

**Figure 1:**
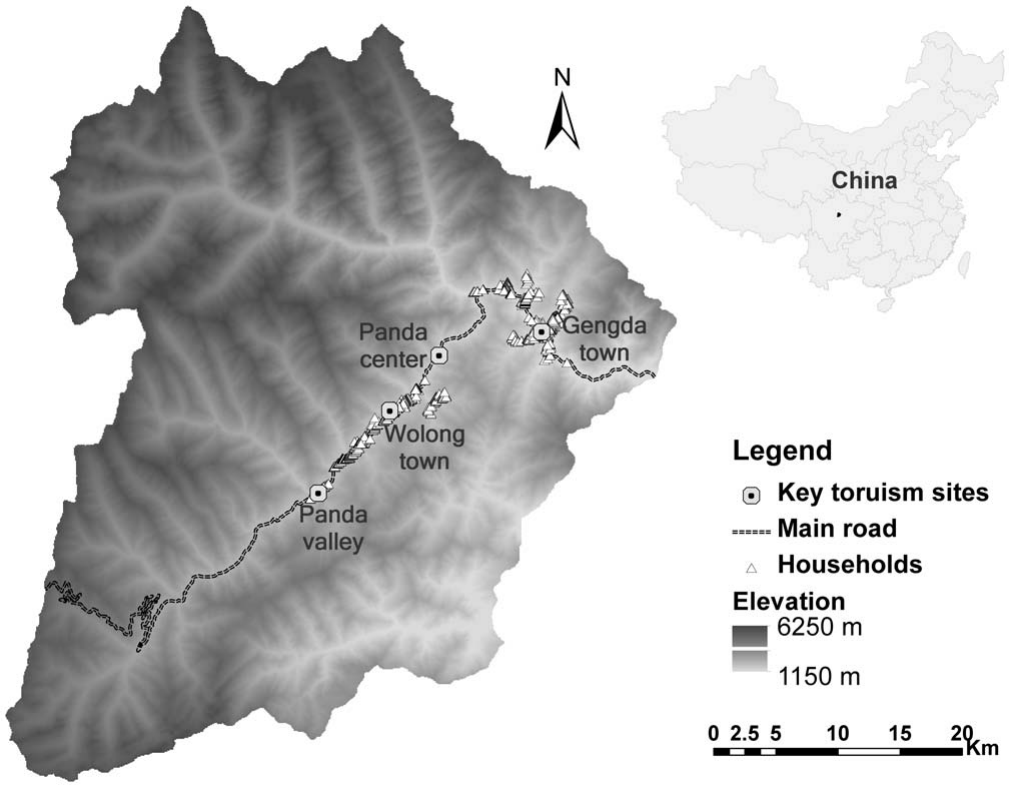
Map of Wolong Nature Reserve, showing its location in China and the distribution of local households and key tourism sites inside the reserve.

Currently, the third most visited country in the world, China's tourism industry is rapidly expanding. Indeed, it is expected to become the world's top tourism destination by 2020 [39]. Tourism development has been practiced in approximately 80% of China's more than 2500 nature reserves, which attracted millions of domestic tourists and a rapidly increasing number of foreign visitors [40], [41]. Wolong Nature Reserve (Figure 1), a flagship protected area in China is among the country's earliest protected areas to develop tourism and is the first national-level nature reserve with an approved ecotourism master plan. Data reported here are from a long-term (since mid-1990s [42], [43], [44]) coupled human and natural system (CHANS, [45]) research project in Wolong Nature Reserve. In this part of the project, we conducted a longitudinal study on a stratified random sample of local households in the reserve from late 1990s to 2007.

Wolong Nature Reserve (102°52′ to 103°24′E, 30°45′ to 31°25′N) is home to the largest wild population of Giant Pandas (*Ailuropoda melanoleuca*), a global conservation icon [46], [47]. The reserve was established in 1963 and expanded to its current size of 2,000 km^2^ in 1975 [48]. Climbing from 1,150 m to 6,250 m in elevation (Figure 1), the reserve hosts hundreds of mammal and avian species and thousands of higher plant species [49], making it part of the Southwestern China Mountains biodiversity hotspot at the global level [50], [51]. The reserve is managed by the Wolong Administration Bureau with two townships under its governance, namely Wolong and Gengda (Figure 1) [52]. In each township there are three villages, each of which is composed of a number of groups. In 2008 there were about 4,600 rural residents distributed in a total of 26 groups. Most local people belong to Tibetan and Qiang ethnic minorities but can speak fluent Mandarin Chinese in a local dialect.

Throughout the twentieth century, local people in this area survived primarily on a subsistence-based agricultural economy that was highly dependent on the natural resources in the reserve. Crop production, livestock-raising, and herbal medicinal plant collection were the most important livelihood strategies of local households [53]. Local people also actively harvested wood, bamboo, and fodder from the forests for daily use. By mid-1990s, annually local community consumed around 10,000 m^3^ of wood for cooking food and pig fodder and heating houses and over 1,000 m^3^ for house construction [44]. At the same time the lack of alternative income also led some local people to pursue poaching and illegal logging [54]. By the end of the century the natural resources extraction activities of local community had resulted in severe destruction of the populations and habitat of wildlife in the reserve, including the giant pandas [42], [43], [44], [55].

In 1979 the reserve became one of China's first three UNESCO biosphere reserves [56]. From then on the conservation issues in the reserve started to receive extensive attention both domestically and internationally. In 1983 the Chinese central government designated the reserve as the nation's first special district for nature conservation, where conservation and development are practiced and managed by the same administrative unit. The Wolong Special District Administration Bureau received direct financial support from the central government and reported to both China's Ministry of Forestry and the Sichuan provincial government. Unprecedented level of funding from the central government helped improve the infrastructure in the area during the 1980s, including the construction of six conservation stations inside and outside the reserve [47]. An international collaboration on panda conservation between China's Ministry of Forestry and World Wildlife Fund (WWF) in early 1980s also resulted in the establishment of the world's largest in-captive panda breeding and research facility in the reserve, which was later named China Center for Research and Conservation of Giant Pandas (Panda center in Figure 1). Between 1984 and 1986 an aid of 887,000 US dollars from World Food Programme (WFP) with a matching fund from China's central government was provided to the reserve to carry out a series of infrastructure construction and forest restoration projects [47].

To stem the ecological degradation in the reserve, various local and national conservation programs were implemented. From mid-1980s to early 2000s, about 550 ha (>70%) of local croplands were reclaimed into tree plantation under a series of local and national payment for ecosystem services programs. Other similar programs were also implemented to pay local households to stop logging, to monitor the forests, and to plant trees in previously logged land [47], [57]. Cropland reduction pushed some households to further diversify their income sources to non-farm activities. These programs also released a large amount of local labors from farming and fuelwood harvesting activities and the labors were also keenly interested in finding non-farm income opportunities.

Tourism has been proposed and adopted as a new development tool to provide alternative income to local farmers and generate additional funds for conservation in the reserve since 1980s [47], [58]. When the earliest tourists came to the reserve to see pandas (mostly caged individuals that were captured from the wild) and their habitat in early 1980s, very few facilities existed and visitors had to live in local government's dorms or local farmers' houses. In early 1990s the Wolong Administration Bureau formed a tourism company, which later became the tourism department of Wolong Administration Bureau. By 1998 two government-owned hotels and several private hotels were built and the annual tourist volume had slowly increased to around 50,000 (Table 1). By mid-1990s, thanks to the continuous success on in-captive breeding at the panda center, the number of new-born pandas increased steadily. Soon the in-captive panda population at the center became the largest in the world. This further enhanced the image of the reserve as the “hometown” of pandas and the panda center became the most important attraction in the reserve [47]. In 1999, a provincial highway linking the reserve to the capital city of the Sichuan province (Figure 1) was completed and greatly improved the accessibility of the reserve to the outside, particularly to group tourists.

**Table 1:** Annual tourist visitation and tourism receipt in Wolong Nature Reserve from 1996 to 2007.

Year	Annual tourist visitation (1000 tourist)	Annual tourism receipts (Million Yuan^a^)	Major events related to tourism development
1996	20.0	No data	
1997	30.4	1.4	
1998	52.4	2.0	The *Wolong Nature Reserve Master Plan* was approved.
1999	66.7	3.2	A provincial highway that connected the reserve to the capital city of Sichuan province was completed.
2000	108.1	12.0	The *Wolong Nature Reserve Ecotourism Master Plan* was approved by the provincial government.
2001	90.0	6.8	
2002	82.0	7.1	The *Wolong Nature Reserve Ecotourism Master Plan* was approved by the central government.
2003	66.0	5.9	SARS outbreak in China severely affected international and domestic tourism.
2004	163.4	29.4	The construction of Wolong Hotel, the only four-star hotel in the reserve, was completed.
2005	206.1	37.1	
2006	235.5	42.4	The Sichuan Giant Panda Sanctuaries World heritage site was officially designated by United Nations Educational, Scientific and Cultural Organization (UNESCO), and a new round of road upgrade construction started.
2007	115.1	20.7	
2008	13.0	No data	The Wenchuan Earthquake (7.9 Mw) struck the reserve on May 12^th^.

a 1 Yuan was equivalent to 0.1200 and 0.1466 US dollars in 1996 and 2008 respectively.

In the early 2000s, the reserve's first ecotourism development master plan was approved by the provincial and central government and the reserve entered a fast tourism development stage. A series of tourism infrastructure development projects, mainly spurred by outside investments, were implemented. For instance, six million dollars were invested to remodel the then largest hotel in the reserve into a new four-star hotel [59]. Dozens of privately owned hotels and restaurants were built by local residents. In 2006, the Sichuan Giant Panda Sanctuary, with Wolong Nature Reserve as a core area, was designated as a World Heritage Site by UNESCO [60]. New road construction was started in the same year to upgrade and widen the main road in the reserve. With these new infrastructure developments providing access and necessary services, tourism in the reserve boomed.

Tourists from around the globe came to the reserve. Seeing pandas in captivity was the top reason for tourists, especially international tourists, to visit the reserve. Many also came to enjoy the forests and the mountainous landscape and to observe wildlife, mainly birds (as mammals, including pandas in the wild, are usually rare and elusive). The Wolong township, where the Wolong Giant Panda Museum, China's first museum for single species, and most of the accommodation facilities were located, hosted the majority of the panda and nature tourists. These tourists usually stayed one to two days and spent almost all their time in key tourism sites and had little interaction with local people, except private hotel owners and managers and souvenir and grocery sellers. While in the other township, Gengda, a different type of tourism started to emerge in 2003. Because of the high elevation, the summer temperature in the reserve is much cooler than that in the nearby cities, every year hundreds of urban residents in Chengdu metropolitan areas came to Gengda township and stayed over a prolonged period in the summer [61]. Most of them chose to stay in local houses or Happy Farmer's Homes (similar to “bed-and-breakfast” operations in Europe and North America [62]). These tourists had more in-depth interactions with their hosts and local neighborhoods.

While overall numbers of tourists and receipts from tourism increased sharply and peaked in 2006 (Table 1), signs of economic leakage were identified in a tourism business survey in 2003–2004 [59]. For instance, most raw produce and meat consumed in local restaurants were purchased from outside vendors instead of local farmers, and a large proportion of high-wage tourism jobs were held by non-local residents. More importantly the largest hotel and the main tourism attractions were operated by an outside investing company. From 1998 to 2006, while the overall tourism receipt in the reserve increased by over 21 fold from about 2.0 million Yuan to about 42.4 million Yuan (Table 1), the rural income from the service sector increased by only seven fold from about 237,000 Yuan to about 1.6 million (calculated based on local government's annual report data). Within the local community, households participating in tourism were located significantly closer to the main road than were non-participating households, indicating a spatial disparity of the distribution of tourism benefits within the reserve [59].

Over the last three decades tourism in the reserve seemed to have followed the model of tourism area life cycle and had gone through the exploration stage in 1980s and involvement stage in 1990s and entered the development stage in 2000s. It was expected that tourism in the reserve would reach a new peak when the road upgrade was completed in early 2008 to welcome the Beijing Olympic Game visitors. However, on 12 May 2008, a devastating (7.9 Mw) earthquake struck the reserve and the surrounding area in Sichuan province. The earthquake and its associated landslides caused extensive damage to the reserve's forests and infrastructure, including the main road and many tourism facilities [63]. A series of reconstruction programs has been implemented to restore the ecological, social and economic systems in the reserve. Tourism has been identified as the primary tool for future economic growth in the reserve and over 200 million US dollars will be spent by Wolong Administration Bureau on tourism infrastructure reconstruction by 2015 [61].

### Data collection

As households are the basic units in which people organize activities such as food and energy consumption, and household members usually make joint or coordinated decisions regarding resource allocation, employment opportunities, and economic production [64], [65], [66], [67], we collected data at the household level.

In 1999, we conducted an initial round of a questionnaire survey to collect baseline information on the socioeconomic status of local households. Because groups are the basic units of human organization in rural China, we conducted in-house personal interviews with a random sample of 220 households (ca. 20% of all households at the time) stratified on all groups in the reserve [42]. Sample households were selected from each stratum (group) with equal probability. Over the past 17 years the research team has had a long-term collaborative relationship with the local government and community. All researchers conducting interviews spoke fluent local dialect and all formal interviews were facilitated by a local assistant so that potential communication error during surveys was minimized. During the interviews we asked household heads or their spouses, who usually had the best knowledge about the household's affairs, about household demographics (e.g., household size, household members' ages, genders, education levels, occupations) and socioeconomic activities (e.g., major income sources, expenditures, energy consumption patterns) in the previous year.

We revisited the sampled households in 2005, 2006, and 2007, the peak tourism development period prior to the earthquake. Besides collecting similar demographic and socioeconomic data as in 1999, we paid special attention to the financial benefits local households received from tourism development. Data required for traditional economic impact analysis are often unavailable in under-developed rural areas because of the lack of reliable accounting/tax systems for small entrepreneurs [68]. Thus, we focused on the type and magnitude of local employment generated directly and indirectly by tourism through recording the main income-generating activities of each member in the households. Direct tourism-related activities included managing (or renting to others to manage) private hotels and/or restaurants; opening Happy Farmer's Homes; working in government-owned tourism hotels or enterprises; driving taxis; and selling souvenirs, food, or other local products to visitors (Table 2). Local households might also earn labor income from temporary infrastructure construction projects or sell local products to hotels, restaurants, shops, or street vendors, and these indirect tourism-related activities were recorded as well (Table 2).

**Table 2:** Number of local rural households receiving different types of direct and indirect financial benefits in the tourism involvement and development stages in Wolong Nature Reserve.

Tourism-related activities	Tourism involvement stage (1998, n = 220)	Tourism development stage (2005–2007, n = 217)
***Direct financial benefits***		
Hotel/Restaurant owners and/or managers	4	11
Leisure farm owners	0	21
Street vendors and souvenir shop owners	5	20
Government-owned hotel employees	0	10
Taxi drivers	0	2
***Sub-total***	***9***	***60*** a
***Indirect financial benefits***		
Working as a temporary infrastructure construction laborer	No data	116
Selling locally collected medicinal herbs	No data	35
Selling locally made honey	No data	29
Selling locally made smoked pork	No data	22
***Sub-total***	***-***	***148*** b
***TOTAL***	***-***	***166***

a Four households participated in more than one type of activity.

b This includes 42 households that received both direct and indirect financial benefits and 106 households that received only indirect financial benefits.

In 2005, we conducted an additional questionnaire survey (Supporting information S1) and asked the interviewees about their general knowledge of the history and status of tourism development in the reserve and to give their personal opinions on a series of 16 questions in four categories: a) their experience of interacting with tourists; b) their perceptions of the socioeconomic benefits of tourism development; c) their perceptions of the various environmental impacts of tourism development; and d) their overall attitudes toward tourism development in the reserve. Interviewees from non-tourism households were also asked to describe specific barriers that prevented them from participating in tourism activities.

The locations of households and of key tourism sites inside the reserve, including two township centers and the entrances of two major tourism attractions (Figure 1), were obtained using a Global Positioning System receiver during the summer of 2006. As travelling inside the reserve is strongly influenced by the high-relief topography and under-developed road system, we chose to compute cost distances instead of Euclidean distances to estimate spatial accessibility of tourism resources to each household using the Path Distance function in ArcGIS 9.3 [69].

### Measurements

In this study, a tourism household was defined as having at least one of its members working on activities directly related to the tourism sector between 2005 and 2007. All other households were classified as non-tourism households. Only tourism households received direct financial benefits from tourism, while both tourism and non-tourism households may have received indirect financial benefits from tourism. The non-financial benefits of tourism were measured based on the interviewees' perceptions of the social benefits of tourism.

We used existing information from the longitudinal survey data to construct household livelihood asset portfolios. Surrogates for all five types of capital were computed (Table 3): a) financial capital - total household income and percentage of nonfarm income (income not from crop plantation or animal husbandry); b) human capital - household size, number of laborers aged between 18 and 49 (in the study area people older than 50 seldom participate in business-related activities), and education level (in years) of the most educated non-student adult in the household; c) natural capital – the amount of cropland owned by a household; d) physical capital – the travel cost distances between households and the nearest key tourism site; and e) social capital - a dummy variable indicating whether a household has kinship relationship (1 = Yes; 0 = No) with government officials and another dummy variable indicating whether a household has kinship relationship (1 = Yes; 0 = No) with village or group heads.

**Table 3:** Basic socioeconomic conditions of the 220 randomly sampled rural households in Wolong Nature Reserve in 1998 and 2006^a^.

Tourism stages	Involvement stage (1998)			Development stage (2006)		
Household type	Tourism	Non-tourism	t test^b^	Tourism	Non-tourism	t test
**Per capita cropland area (in Mu** c **)**	1.97 (0.87)^d^	2.61 (1.56)	3.74 ***	0.63 (0.41)	1.25 (0.93)	6.73 ***
**Per capita income (in Yuan)**	1992 (1733)	1327 (1494)	2.03 **	6429^e^ (5068)	5157 (6323)	1.31 *
**Nonfarm income %**	40.7% (32.2%)	36.3% (31.4%)	1.03	66.2% (29.3%)	37.9% (29.6%)	4.27 ***
**Poverty rate** f **%**	35.00%	35.85%	NA	0	3.23%	NA

a The overall response rates in 1998 for cropland and income questions were 95.5% and 99.1%, respectively, and those in 2006 were 87.6% and 84.8%, respectively.

b Student's t test was used to compare cropland and income between tourism and non-tourism households. The signs *, ** and *** represent significance at 10%, 5%, and 1% levels respectively.

c 1 Mu = 0.0667 Ha.

d Standard deviation is shown in parentheses.

e The income measurements in tourism development stage have been inflation-adjusted.

f Standard rural poverty lines published by Chinese government in 1999 and 2006 [80], [81] are used.

### Data analysis

We used logistic regression procedures to estimate parameter values in multivariate models of household-level tourism participation. Logistic regression is an appropriate statistical technique for analyzing models of dichotomous dependent variables. We report parameters from the logistic regression equations in the form:
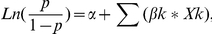
where p is the probability that a household participates directly in tourism activities, p/(1−p) is the odds of tourism participation, α is a constant term, β_k_ represents the effect parameter of the explanatory variables, and X_k_ represents the explanatory variables in the model, which include livelihood asset variables and township as a contextual factor. Coefficients in a logistic model give the change in the log-odds of tourism participation for a unit change in the explanatory variables. To facilitate interpretation of the coefficients, we report the odds ratios, which are interpreted as the amount by which the odds of tourism participation are multiplied for each unit change in the explanatory variable. Odds ratios equal to 1 represent no effect; odds ratios greater than 1 represent positive effects; and odds ratios less than 1 represent negative effects.

To estimate the accuracy and reliability of the model we conducted a ten-fold cross validation [70]. The samples were randomly divided into ten subsets (half composed of 21 households and the other half composed of 22 households). We iteratively (i.e., ten times) used nine subsets to train the model and the remaining to validate it. In each iteration we generated a receiver operating characteristic (ROC) curve and calculated the area under the ROC curve (AUC) as a measure of model accuracy.

We further examined how household-level tourism participation might affect local residents' perceptions and attitudes toward tourism development in the reserve. Because local households' choice of participating in tourism was not the result of a randomized or natural experiment, systematic differences between tourism and non-tourism households may constitute confounding effects, thus making it spurious to estimate the effects of household-level tourism participation on the interviewees' perceptions/attitudes. The self-selection nature of tourism participation creates a counterfactual question – “what would be the perception/attitude of a person in a tourism household if his/her household were not directly participating in tourism?” Ignoring this issue may lead to invalid inferences [71], [72].

We approached this issue with a propensity score weighting methodology [71], [72]. A propensity score is the conditional probability of receiving the treatment given the observed covariates [72]. The logic is that we may make causal inferences if we compare individuals in the treatment group (in our case, respondents from tourism households) to those in the control group (respondents from non-tourism households) with similar propensity scores. The propensity score is defined as [72]:
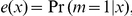
where m is a dummy variable indicating the treatment (i.e., 1 for tourism household and 0 for non-tourism household); and e(x) is the propensity for receiving the treatment, which can be estimated from a logistic regression. We then used an inverse probability of treatment weighting method to estimate the average causal effect of household tourism participation on respondents' perceptions and attitudes [71], [73]. The weights are determined by:
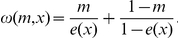



Therefore, a tourism household is weighted by 1/e(x) and a non-tourism household is weighted by 1/(1−e(x)). In this way, more weight is assigned to a tourism household with a lower propensity score and to a non-tourism household with a higher propensity score, such that the estimation of the average causal effect focuses mainly on the strongest overlap in propensity between the two groups. The weight is then used in a series of weighted linear regressions (for Likert-type scale questions in categories b, c, and d) and weighted logistic regressions (for Yes/No questions in category a). In addition to the household-level participation in tourism, we controlled for the household's locations (township and travel cost distance to key tourism sites) and social ties and the respondent's age, education, gender, and occupation as covariates in these regression models (see detailed descriptive statistics of these control variables in Supporting Information Table S1 ).

All statistical modeling and analyses were conducted using PASW Statistics 18, Release Version 18.0.0 (SPSS, Inc., 2009, Chicago, IL, www.spss.com). Significance levels were set at 0.05, 0.01, and 0.001.

## Results

### Direct and indirect financial benefits of tourism received by local households

In 1998, nine (4%) out of the 220 households sampled directly participated in tourism-related activities, with four owning private hotels and five selling souvenirs (Table 2). The number of tourism households increased to 60 (28%) in the peak tourism development period (2005–2007) before the earthquake. A total of 83 individuals from these 60 households worked in tourism-related jobs and 52 of them (62.7%) were females. In other words, by mid-2000s about 9.1% of the sampled population (896 individuals in 217 households) had worked in the tourism industry.

During the peak tourism development period (2005–2007) many local households also received indirect financial benefits from tourism development (Table 2). For instance, a total of 116 households claimed to have received some income from temporary labor jobs on infrastructure construction inside the reserve (primarily road construction), 87 of which were non-tourism households. A number of households also claimed to have earned income from selling medicinal herbs (14 tourism households and 25 non-tourism households), honey (6 tourism households and 19 non-tourism households), and smoked pork (10 tourism households and 12 non-tourism households) that were collected or made locally. Most of these local products were sold to local restaurants, shops, and street vendors, which eventually were purchased by outside visitors. Fifty-one (23.5%) households received neither direct nor indirect income during the peak tourism development stage.

Changes in the basic socioeconomic status of the randomly sampled households from the tourism involvement stage (late 1990s) to the peak development stage (2005–2007) are listed in Table 3. In 1998, the 60 households who were later classified as tourism households had on average less cropland and more income per capita than the other households had. More than two-thirds of the reserve's croplands were reclaimed to tree plantation between 2000 and 2003. On a per capita basis, both types of households reclaimed about the same amount of cropland and received similar monetary subsidies from the two PES programs. By 2006, the mean per capita income of both groups increased significantly, and the net difference in per capita income (inflation adjusted) between the two types of households almost doubled to 1300 Yuan (∼166 US dollars, 1 Yuan was equivalent to 0.1280 US dollars as of Dec. 2006). Non-tourism households generally earned more farm income by replacing subsistence crops (e.g., corn and potato) with cash crops (e.g., cabbage and turnips), while their mean non-farm income percentage remained at around 36 to 38% from the late 1990s to mid-2000s. Direct and indirect tourism income was most important to tourism households, and their mean non-farm income percentage increased from 40% to over 66% between the late 1990s and mid-2000s.

### Determinants and barriers of household-level participation in tourism

A final sample of 215 households was included in the logistic regression model after excluding two households whose income data in the tourism involvement stage were incomplete and three households that were not present in 2005 (due to death or emigration). The binomial logistic regression model on household tourism participation includes 11 independent variables, the descriptive statistics of which are listed in Table 4. Ninety-four of the sampled households were located in Wolong township and the other 121 were Gengda residents. Then mean household size was about 4.1. The mean number of laborers was around 1.7. The most educated non-student adult in the household received on average 7.7 years of education. Each household owned an average of 3.9 Mu (1 Mu = 0.067 ha) cropland. The mean annual household income in 1998 was 8,059 Yuan (∼973 US dollars, 1 Yuan was equivalent to 0.1208 US dollars in 1998) and the mean non-farm income percentage in 1998 was 38%. The number of households with government- and village-level social ties was 24 (11.2%) and 39 (18.1%), respectively.

**Table 4:** Results of the binary logistic regression model on household-level tourism participation (n = 215).

Variable	Description	Mean (SD)	Parameter^a^ (Robust SE)	Odds Ratio
***Financial capital***				
Log(Income 98)	Log transformed total household income in 1998 (in Yuan)	8.3319 (0.9171)	0.6799 * (0.3392)	1.9737
Nonfarm income%	Percentage of nonfarm income in total income in 1998	0.3756 (0.3164)	−0.9945 (0.8140)	0.3699
***Human capital***				
Household size	Number of people in each household	4.1302 (1.4115)	0.1748 (0.1732)	1.1910
Education	Education level (in years) of the most educated non-student adult in the household	7.7023 (3.289)	0.2161 ** (0.0836)	1.2413
Labor	Number of labors	1.6698 (1.0402)	0.7239 * (0.3264)	2.0625
***Natural capital***				
Cropland	Total cropland acreage of the household (in Mu)	3.8544 (2.4621)	−0.3943 *** (0.1101)	0.6742
***Physical capital***				
Log(Cost distance)	Log-transformed cost distance between the household and the nearest key tourism site	8.8583 (1.0088)	−0.8862 *** (0.2771)	0.4122
***Social capital***				
Tie_Government	Whether the household has a member or mmediate relative working in local government: 1. Yes; 0. No	0.1116 (0.3156)	2.2067 ** (0.7792)	9.0855
Tie_Village	Whether the household has a member or immediate relative being a village or group head: 1. Yes; 0. No	0.1814 (0.3862)	1.0820 * (0.5015)	2.9507
***Contextual factor***				
Township	1. Wolong township; 0. Gengda township	0.4372 (0.4972)	0.8423 * (0.4200)	2.3216
Intercept			−2.1448 (3.9749)	0.1171
Wald χ^2^			45.0600 ***	
Log-Likelihood			−76.6641	
Pseudo R^2^ (Nalgelkerke)			0.5410	
Ten-fold cross validation prediction accuracy			87.88%	
Ten-fold cross validation AUC			0.9338	

a The signs *, **, and ***, represent significance at the 5%, 1%, and 0.1% levels, respectively.

All five categories of capital seem to influence the likelihood of household-level participation in tourism. The annual household income in 1998 (financial capital) was a significant explanatory variable and higher income in tourism involvement stage increased the odds of tourism participation (p<0.05), but the non-agriculture income percentage in 1998, an indicator of the household's economic reliance on non-farm income opportunities before the tourism boom, was not significant. In terms of human capital, households with more laborers were significantly more likely (p<0.05) to be involved in tourism with each additional laborer increased the odds of household tourism participation by 2.06. Education had a positive effect on the likelihood of the household's participation in tourism (p<0.01), but household size did not. Households with more cropland (natural capital) tended not to participate in tourism (p<0.001). The more it cost physically to travel between a household and the closest key tourism site (physical capital), the less likely (p<0.01) the household would participate in tourism. A household's social capital has some influence on its likelihood to take part in tourism. Having a kinship relationship with government officials and village or group heads increased the odds of tourism participation by nine times (p<0.01) and three times (p<0.05), respectively. Township was also a significant predictor since households located in Wolong township, where the main tourism attractions were located, were significantly more likely (p<0.05) to participate in tourism than households in Gengda township.

In 2005, households were revisited and the heads or their spouses were asked a series of questions on their knowledge and perceptions of tourism development in the reserve. A total of 192 households answered the questions, including 55 tourism households and 137 non-tourism households. When the non-tourism household heads (or their spouses) were asked about what prevented their household members from participating in tourism, a variety of barriers were reported. Financial and physical limitations were mentioned most often, including lack of start-up funds (60.1%), household location being far from key tourism sites (57.5%), and lack of land and housing to start a tourism business (27.5%). Fifteen interviewees (10.1%) stated that the lack of transparent and supportive local tourism policies made them feel uncertain about the economic potential of tourism development. Other respondents referred to human and social capitals, such as the lack of social connections (9.4%), the lack of labor (6.5%), being too old to have a business (5.1%), and the lack of experience (4.3%). These responses are consistent with the logistic model results on determinants of household-level tourism participation.

### Non-financial tourism benefits perceived by local households

We measured tourism's non-financial benefits on the basis of local people's perceptions in 2005. The interviewees were asked about how they interacted with tourists. Being in a tourism household increased the odds of communicating with tourists and receiving information about job opportunities from tourists by 7.17 (p<0.001) and 3.44 times (p<0.001), respectively (Table 5). In contrast, the odds ratios were 5.78 (p<0.01) and 2.82 (p<0.01), respectively, in the unweighted models. The respondents also reported other types of information exchange with tourists. For instance, from tourists they received information about tourism development and policies in other areas, about the tourists' experiences and impressions about the reserve, and about the tourists' suggestions to improve tourism services. In return, they provided information to tourists on local wildlife distribution (especially pandas), culture, and conservation issues. Over one-third of the interviewees acknowledged there had been conflicts between locals and tourists (Table 5). They reported that conflicts usually took place during bargaining between local souvenir sellers and tourists or when some Happy Farmer's Homes (HFH) tourists filched vegetables from households' cropland.

**Table 5:** Estimated effects of household-level tourism participation on local residents' interactions with tourists.

	% agreed^a^		Coefficients^b^ (SE) [Odds ratio]	
Household type	Tourism	Non-tourism	Weighted	Unweighted
1. I have had some communications with tourists.	73.6%	40.7%	1.0375 *** (0.2722) [7.1654]	1.2363 ** (0.4090) [5.7836]
2. I have received information about job opportunities from tourists.	19.2%	6.8%	1.7550 *** (0.5144) [3.4430]	1.9693 ** (0.6723) [2.8222]
3. There have been conflicts between local residents and tourists.	40.5%	32.6%	−0.5042 (0.3140) [0.8930]	−0.1132 (0.4370) [0.6040]

a The sample sizes for tourism and non-tourism households are 53 and 135 (Q1–2) and 42 and 92 (Q3), respectively.

b The signs ** and *** represent significance at the 1% and 0.1% levels, respectively.

Residents perceived socioeconomic benefits occurring to their households from tourism (Table 6). Almost everyone interviewed agreed that tourism development improved public services and living conditions, enhanced most families' quality of life, and built a good image of the reserve among outside people. Tourism household members tended to agree more (p<0.05) with the statement “tourism development has helped enhance my family's quality of life”. They tended to agree less with two other statements “tourism development has helped enhance most families' quality of life across the reserve” (p<0.05 in the weighted model and p>0.10 in the unweighted model) and “tourism development has helped to build a good image of the area among outside people” (p<0.01 in weighted model and p<0.05 in unweighted model).

**Table 6:** Estimated effects of household-level tourism participation on local residents' perceptions^a^ on the socioeconomic benefits of tourism development.

	Mean score^a^^,^ b (SD)		Coefficients^c^ (SE)	
Household type	Tourism	Non-tourism	Weighted	Unweighted
1. Tourism development has helped improve public service and living environment.	1.74 (0.64)	1.70 (0.70)	−0.0904 (0.0857)	−0.0925 (0.1082)
2. Tourism development has helped enhance my family's quality of life.	0.64 (1.73)	−0.58 (1.73)	0.6956 * (0.2703)	0.8098 * (0.3143)
3. Tourism development has helped enhance most families' quality of life in the reserve.	1.56 (0.79)	1.69 (0.63)	−0.2522 * (0.1047)	−0.1565 (0.1094)
4. Tourism development has helped to build a good image of the area among outside people.	1.52 (0.72)	1.60 (0.72)	−0.2825 ** (0.0931)	−0.2644 * (0.1104)

a Five-point Likert scale: −2. Strongly disagree; −1. Disagree; 0. Neutral; 1. Agree; 2. Strongly agree.

b The sample sizes for tourism and non-tourism households are 55 and 136 (Q1–3) and 52 and 125 (Q4), respectively.

c The signs * and ** represent significance at the 5% and 1% levels, respectively.

### The influence of tourism participation on local residents' environmental awareness

Local people's perceptions of the environmental impacts of tourism development are listed in Table 7. In general, respondents from all households perceived almost no negative impact on the air and water quality, the soundscape (i.e., the natural acoustic environment), the mountain trails, and the natural forests in the reserve; the perceived a low level of negative impacts on wildlife including pandas (e.g., hikers disturbing wildlife) and the availability of medicinal herbs (e.g., tourists collecting some specific herbs in the reserve); and they perceived a medium level of negative impact on road traffic (e.g., increasing traffic congestion and accidents). People from tourism households tended to perceive significantly higher levels of negative impacts on wildlife (p<0.01 in the weighted model and p<0.05 in the unweighted model) and road traffic (p<0.05 in the weighted model and p>0.10 in the unweighted model) than those from non-tourism households, and the influences of household-level tourism participation on other environmental impact perceptions were not significant. Overall, while almost all households acknowledged that tourism was good for the reserve, being in a tourism household seemed to make people disagree less with the statement that “there are conflicts between tourism development and conservation in the reserve” (p<0.01) (Table 8).

**Table 7:** Estimated effects of household-level tourism participation on local residents' perceptions a on the direct negative environmental impacts of tourism development.

	Mean score (SD)		Coefficients^b^ (SE)	
Household type	Tourism (n = 55)	Non-tourism (n = 137)	Weighted	Unweighted
Air and water quality	0.02 (0.13)	0.01 (0.09)	0.0069 (0.0137)	0.0148 (0.0188)
Soundscape	0.11 (0.46)	0.04 (0.22)	0.0855 (0.0516)	0.0810 (0.0580)
Road traffic	1.67 (0.84)	1.56 (0.80)	0.2490 ^*^ (0.1208)	0.2193 (0.1346)
Mountain trail	0	0.01 (0.12)	−0.0056 (0.0133)	−0.0033 (0.0190)
Natural forest	0.05 (0.30)	0.04 (0.27)	0.0175 (0.0498)	0.0077 (0.0517)
Medicinal herbs	0.42 (0.79)	0.39 (0.70)	0.1959 (0.1320)	0.0206 (0.1480)
Wild pandas and other wildlife	0.31 (0.66)	0.03 (0.21)	0.1918 ** (0.0631)	0.1848 * (0.0750)

a 0 = No impact, 1 = Low level, 2 = Medium level, 3 = High level. No positive impact was reported.

b The signs * and ** represents significance at the 5% and 1% levels, respectively.

**Table 8:** Estimated effects of household-level tourism participation on local residents' overall attitudes toward tourism development.

	Mean score^a^^,^ b (SD)		Coefficients^c^ (SE)	
Household type	Tourism	Non-tourism	Weighted	Unweighted
1. There are conflicts between tourism development and conservation in the reserve.	−0.54 (1.66)	−1.37 (1.17)	−0.6143 ** (0.2271)	−0.6382 ** (0.2654)
2. Overall tourism development is good for the reserve.	1.85 (0.49)	1.95 (0.28)	−0.0819 (0.0508)	−0.0866 (0.0656)

a Five-point Likert scale: −2. Strongly disagree; −1. Disagree; 0. Neutral; 1. Agree; 2. Strongly agree.

b The sample sizes for tourism and non-tourism households are 46 and 114 (Q1) and 55 and 134 (Q2), respectively.

c The signs ** represents significance at the 1% level.

## Discussion

Despite the high level of overall economic leakage reported in a previous study [59] and in this study, tourism development in Wolong Nature Reserve before the 2008 earthquake generated a broad range of economic and social benefits to the local community. First, over three-quarters of the sampled households received more or less financial benefit directly or indirectly from tourism. There are likely also other economic benefits not captured in our measurements, as tourism is a diverse industry with the potential to support other economic activities through creating income opportunities throughout a complex supply chain of goods and services. For example, while many tourism jobs were taken by outsiders [59], they consumed a significant amount of local produce and spent money in local restaurants and shops. Another interesting finding is that there were more female local residents than males working in the tourism industry. This confirmed tourism's potential to promote gender equity in developing countries [74]. Second, tourism development improved the infrastructure and living conditions of the community, especially through construction and upgrading of the main road. The road greatly facilitated the sales of cash crop (e.g., cabbage and turnip) to the outside market, which constituted a major income source for the majority of the rural households. This was well recognized by the interviewees. Moreover, tourism provided opportunities for local people to communicate with outsiders.

Nevertheless, the overall magnitude of economic benefit that local community received from tourism was yet limited and there was still disparity in the tourism-derived benefit distribution within the local community. Slightly over one-quarter of our sampled households earned some income directly from tourism. This direct tourism income was not the most important source of income for most households, except those who owned or managed a year-around hotel or restaurant (∼5%). At a reserve level in 2006, income from the service sector represented only 7.4% of the total rural economic income in the two townships [75]. Our binomial logistic regression model results revealed that the quantity and quality of the various capital possessed by a household determined whether it had the capability and motivation to pursue tourism as a new livelihood strategy. Households with less natural capital to earn on-farm income in this reserve tended to have more pressure to find income opportunities from tourism, which was one of the very limited non-farm alternatives in the reserve. Plentiful financial capital made a household capable of making necessary investments (e.g., builds a private hotel, purchase a car) to participate in tourism and the lack of such capital was mentioned by many non-tourism households as a major barrier to participating in tourism industry. Human capital was shown to matter. First, households with better-educated adults tended to benefit more from tourism, as they might possess better skills (e.g., the ability to communicate with outsiders, knowledge of language beyond the local dialect) for participation in tourism or a better ability to acquire such skills. Second, households with more adult laborers have greater pressure to find non-farm income opportunities to make use of the surplus labor. Social capital, especially a household's kinship with government employees, was an important predictor of tourism participation. Households having close relationships with township- and reserve-level government officials were in a better position to acquire tourism-related information and critical resources (e.g., loan opportunities). This is consistent with previous findings in this reserve. Earlier evidence showed that almost all non-rural small tourism business managers were a relative of local government officials [59]. Physical capital, measured as a household's proximity to the closest key tourism site, also influenced the likelihood of participation in tourism, because tourism income opportunities were found to be disproportionally distributed around those locations.

Our results showed households receiving more direct financial benefits tended to perceive more non-financial benefits. They tended to communicate more with tourists and exchange information with tourists; and they perceived more positive impacts of tourism on their standards of living. Despite some minor conflicts reported, the advantages of tourist-resident contact seem to outweigh the disadvantages, because such communications may help to break the feeling of isolation of rural minorities and visitors in the reserve, create mutual awareness of each group, and provide an opportunity to learn from each other. Such contact can be a starting point for more fundamental inter-cultural encounters, through which the educational potential of nature-based tourism can be realized. As these financial and non-financial benefits accrue faster to some tourism households than others, the existing disparity in the livelihood assets between tourism and non-tourism households may increase. This may further augment social and economic differentiation within the community.

Besides the socioeconomic benefits to local residents, nature-based tourism also has the potential to enhance the environmental awareness and attitudes of local residents [9], [37], [76]. After several years of tourism development, we observed a high degree of agreement among respondents with regard to the positive socioeconomic impacts of tourism in the reserve. During the interviews, all interviewees acknowledged that pandas and forests are the top tourism attractions of the reserve. Thus those who participated in and benefited from tourism became more aware of the link between the economic value of natural ecosystems and conservation success. Despite their very favorable disposition towards tourism development, some respondents, especially those in tourism households, recognized that some types of negative environmental impacts may ensue. People from tourism households tended to be more knowledgeable about the intensities and the spatial distributions of tourists' activities through their interactions with tourists. Because they derived direct tourism benefits from the conservation of pandas and other wildlife, they were more likely to care about the ecosystem that harbored them. This increased awareness may help explain why more respondents from tourism households tended to think that there were conflicts between tourism development and conservation in the reserve. Overall, these are all signs that tourism development may positively influence the environmental awareness and attitudes of the local people, which in the long run may enhance local people's conservation behaviors.

From a policy perspective, the experience learned from past tourism development in Wolong Nature Reserve is of great value for making relevant interventions in the future. The 2008 earthquake, which reset the tourism development in the area, offers an opportunity for the reserve to develop tourism that may better benefit the poor. The post-earthquake reconstruction plan includes a new round of local household relocations from remote mountainous areas to roadside areas, with their cropland being reclaimed for tree and bamboo plantation as one way to restore more habitats for the giant panda and other wildlife species. To construct new tourism facilities in the Gengda township, a significant amount of cropland was requisitioned with cash compensation to the affected households. While growing cash crop still constitutes an important and stable income source of many households, those who had to trade their cropland with cash compensation will inevitably be facing more limited livelihood options in the future. In the short run, many households may earn wage-labor income from the ongoing infrastructure reconstruction projects. But after the completion of reconstruction, as tourism has been identified as the major economic development tool in the reserve and the surrounding region, the importance of tourism-related income for the local households will be even greater in the future than before the earthquake. The Wolong Administration Bureau needs to design and implement policies to improve local households' capacities to pursue tourism as a major livelihood strategy. On the one hand, policies that specifically target the poor and help augment their livelihood assets (e.g., provide training to enhance human capital and making loan opportunities accessible to enhance financial capital) are needed. On the other hand, other regulations that encourage tourism operators to transfer significant amounts of benefits to the poor are also needed. For example, the government may require outside tourism operators or developers to preferentially provide job opportunities to people from the poorer households, rather than letting nepotism prevail as it has in the past [59].

Perhaps more importantly, involvement and integration of local communities into the entire tourism development process is critical for achieving ecological and socioeconomic sustainability in protected areas [77]. Thus, local people, especially the poor, should be included in the policy design process from the very beginning. This is specifically relevant to countries like China, where conservation programs are usually implemented in a top-down manner with little input from the local stakeholders [78], [79]. In the past, although there were two reserve-wide tourism stakeholder meetings organized by the Wolong Administration Bureau in 2001 and 2007, besides related government officials, only tourism business owners were invited (W. Liu, personal observation). The consequence was that most local people were only aware of the existence of tourism policies but not the details, which had prevented some capable households from participating in tourism, as reported by some respondents in our interviews. We suggest that local government first needs to expand their tourism stakeholder list to include all community members with willingness/interest to participate in tourism, carefully listen to their suggestions and understand their needs, and then design policies and regulations that will give poorer members priorities to participate in tourism and benefit from it. In the long run, to sustain a high-level of local participation in tourism, the current top-down decision-making, implementation, and management style in tourism development has to be changed to a multistakeholder-based, horizontal one.

Last but not least, our results highlight the strength of longitudinal data and quantitative analysis in understanding the impacts and effectiveness of nature-based tourism and ICDPs in general. While the need to conduct environmental monitoring of nature-based tourism is well recognized [30], the importance of monitoring socioeconomic changes is often overlooked, as is understanding the drivers behind the changes. By documenting the specific changes on the types and levels of tourism participation and the characteristics of community members, we may establish more precisely the contexts that give rise to the observed impacts. Limited by time and monetary costs, after-the-fact analyses or simulation are more often used in impact assessment, but monitoring changes across time, particularly early to tourism growth stages, can accumulate data not possible to acquire by other methods and produce information with higher degrees of managerial utility and policy relevance. We suggest that socioeconomic impact measurement and change monitoring must be firmly incorporated into nature-based tourism planning and management in protected areas of developing countries from the early phases of development. Meaningful local involvement can then be ensured and positive impacts on poverty reduction and conservation can be effectively promoted.

## Supporting Information

Supporting Information S1 **The questionnaire on local residents' perceptions and attitude toward tourism development in Wolong Nature Reserve, China.**
(DOC)

Table S1 **Descriptive statistics of the household and individual level variables in estimating the effects of household-level tourism participation on local residents' perceptions.**
(DOC)
