# Supplementary material for: Drivers and Socioeconomic Impacts of Tourism Participation in Protected Areas
Source: PLoS One. 2012 Apr 25;7(4):e35420. doi: 10.1371/journal.pone.0035420 (PMC3338832; doi:10.1371/journal.pone.0035420)
Supplement: Supporting Information S1 — The questionnaire on local residents' perceptions and attitude toward tourism development in Wolong Nature Reserve, China. (DOC) [file pone.0035420.s001.doc]

### S1. Questionnaire

QUESTIONAIRE ON LOCAL RESIDENTS’ PERCEPTIONS AND ATTITUDE

TOWARD TOURISM DEVELOPMENT IN WOLONG NATURE RESERVE

Interviewer: ______ Date: ______ Time: ______

Interviewee information

Household ID: _____ Township: ______ Village: ______ Group: _____

Interviewee’s relationship with household head: ________________

Birth year: ____ Gender: _____ Ethnicity: _____ Education: __

Other information: ________________________________________________________

Questions:

1. What is your general feeling about the tourism development plan in the reserve?

 It is promising  It is not promising

 It is hard to tell  I don’t know the plan

2. In the past, in which year did you observe the highest volume of tourists in the reserve?

 Before 2000  2000  2001  2002

 2003  2004  2005  I have no idea

3. What are the peak tourism months in the reserve? _____________________________

4. (For household participating in tourism activities) How do you think the tourism development plan will affect your business?

 It will positively affect my business

 It will negatively affect my business

 I have no idea about how it will affect my business

5. (For household not participating in tourism activities) what have prevented your household from participating in tourism activities?

6. Based on your knowledge, what are the tourists coming to visit the reserve for?

 Giant panda  Mountainous landscape and scenery

 Comfortable climate  Local product

 Other: ______________

7. Based on what you have observed, which areas in the reserve do tourists go？

 Dengsheng  HetaoPing  LaoyaShan

 QicenglouGou  ShaWan  WuyiPeng

 XingfuGou  YingchangGou  YingxiongGou

 ZhengHe  Others ______

8. Please, based on your best knowledge, rate the environmental impacts that have been caused by tourism development n the reserve.

| Categories | High level | Medium level | Low level | No impact | Comments |
| --- | --- | --- | --- | --- | --- |
| Air and water quality |  |  |  |  |  |
| Soundscape |  |  |  |  |  |
| Road traffic |  |  |  |  |  |
| Mountain trail |  |  |  |  |  |
| Natural forest |  |  |  |  |  |
| Medicinal herbs |  |  |  |  |  |
| Wild pandas and other wildlife |  |  |  |  |  |

9*. I have had some communications with tourists.

 Strongly disagree  Disagree  Neutral  Agree  Strongly agree

 I don’t understand the statement

Comments: ___________________________________________________________

10*. I have received information about job opportunities from tourists.

 Strongly disagree  Disagree  Neutral  Agree  Strongly agree

 I don’t understand the statement

Comments: ___________________________________________________________

11*. There have been conflicts between local residents and tourists.

 Strongly disagree  Disagree  Neutral  Agree  Strongly agree

 I don’t understand the statement

Comments: ___________________________________________________________

12. Tourism development has helped improve public service and living environment.

 Strongly disagree  Disagree  Neutral  Agree  Strongly agree

 I don’t understand the statement

Comments: ___________________________________________________________

13. Tourism development has helped enhance my family's quality of life.

 Strongly disagree  Disagree  Neutral  Agree  Strongly agree

 I don’t understand the statement

Comments: ___________________________________________________________

14. Tourism development has helped enhance most families' quality of life in the reserve.

 Strongly disagree  Disagree  Neutral  Agree  Strongly agree

 I don’t understand the statement

Comments: ___________________________________________________________

15. Tourism development has helped to build a good image of the area among outside people.

 Strongly disagree  Disagree  Neutral  Agree  Strongly agree

 I don’t understand the statement

Comments: ___________________________________________________________

16. There are conflicts between tourism development and conservation in the reserve.

 Strongly disagree  Disagree  Neutral  Agree  Strongly agree

 I don’t understand the statement

Comments: ___________________________________________________________

17. Overall tourism development is good for the reserve.

 Strongly disagree  Disagree  Neutral  Agree  Strongly agree

 I don’t understand the statement

Comments: ___________________________________________________________

*. The answers in these questions were reclassified into binary classification in data analysis (Yes: “Strongly agree” and “Agree”; No: “Strongly disagree” and “Disagree”; No data: “Neutral”)

### 
