# Supplementary material for: Drivers and Socioeconomic Impacts of Tourism Participation in Protected Areas
Source: PLoS One. 2012 Apr 25;7(4):e35420. doi: 10.1371/journal.pone.0035420 (PMC3338832; doi:10.1371/journal.pone.0035420)
Supplement: Table S1 — Descriptive statistics of the household and individual level variables in estimating the effects of household-level tourism participation on local residents' perceptions. (DOC) [file pone.0035420.s002.doc]

### Table S1. Descriptive statistics of the household and individual level variables in estimating the effects of household-level tourism participation on local residents’ perceptions.

| **Variables** | **Description** | **Mean ^a^** | **SD** |
| --- | --- | --- | --- |
| ***Household-level*** | | | |
| Tourism household | 1. Yes; 0. No | 0.2865 | 0.4533 |
| Township | 1. Wolong township; 0. Gengda township | 0.3979 | 0.4908 |
| Log(Cost distance) | Log-transformed cost distance between the household and the nearest key tourism site | 8.8535 | 1.0408 |
| Tie_Government | Whether the household has a member or immediate relative working in local government - 1. Yes; 0. No | 0.1146 | 0.3194 |
| Tie_Village | Whether the household has a member or immediate relative being a village or group head - 1. Yes; 0. No | 0.1875 | 0.3913 |
| **Individual-level** | | | |
| Gender | 1. Female; 0. Male | 0.3229 | 0.4688 |
| Education | Number of years of formal education that the respondent received | 4.6927 | 3.6901 |
| Age | Age of the respondent (in years) | 47.4219 | 13.1585 |
| Occupation | The main occupation of the respondent - 1. Farmer; 0. Others | 0.8125 | 0.3913 |

a. n=192, one interviewee in each household, including 55 tourism households and 137 non-tourism households.
